# Supplementary material for: Limited effects of m6A modification on mRNA partitioning into stress granules
Source: Nat Commun. 2022 Jun 29;13:3735. doi: 10.1038/s41467-022-31358-5 (PMC9243116; doi:10.1038/s41467-022-31358-5)
Supplement: Supplementary file 1 — Supplementary Information [file 41467_2022_31358_MOESM1_ESM.pdf]

Supplementary Figure 1

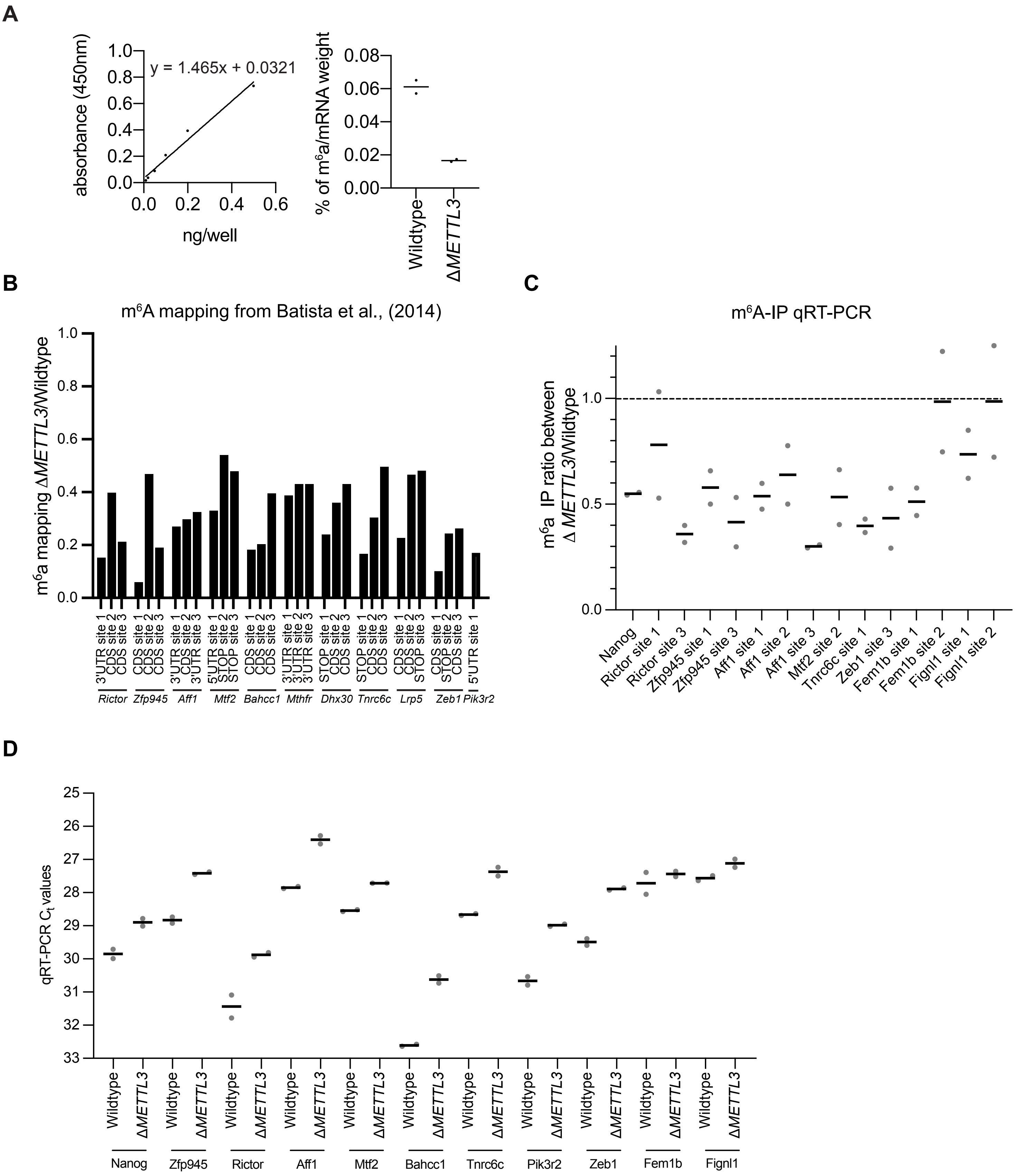

Supplementary Figure 1. Δ*METTL3* mES cells is m^{6}A-depleted for many RNAs that are inspected by smFISH partitioning in stress granules. (A) Left graph: OD450 absorbance values from m^{6}A standard controls provided by EpiQuick m^{6}A RNA Methylation Quantification Kit was measured and a linear regression was plotted to quantify the absolute amount of m^{6}A in mRNA isolated from wildtype and Δ*METTL3* mES cells shown in the right graph. Right graph: Fraction of m^{6}A weight relative to all nucleic acid weight in mRNAs between wildtype and Δ*METTL3* mES cells. (B) Fold change differences in the m^{6}A-immunoprecipitation of individual m^{6}A sites from 11 mRNAs (Δ*METTL3* mES cells/wildtype cells). (C) m^{6}A-immunoprecipitation qRT-PCR ratio between Δ*METTL3* and wildtype mES cells for 15 m^{6}A-mapped sites. Two biological replicates were performed. Line indicates the median value. (D) qRT-PCR Ct values for wildtype and Δ*METTL3* for multiple m^{6}A modified RNAs. Two biological replicates were performed. Line indicates the median value. Source data are provided for this Figure (see data availability).

Supplementary Figure 2

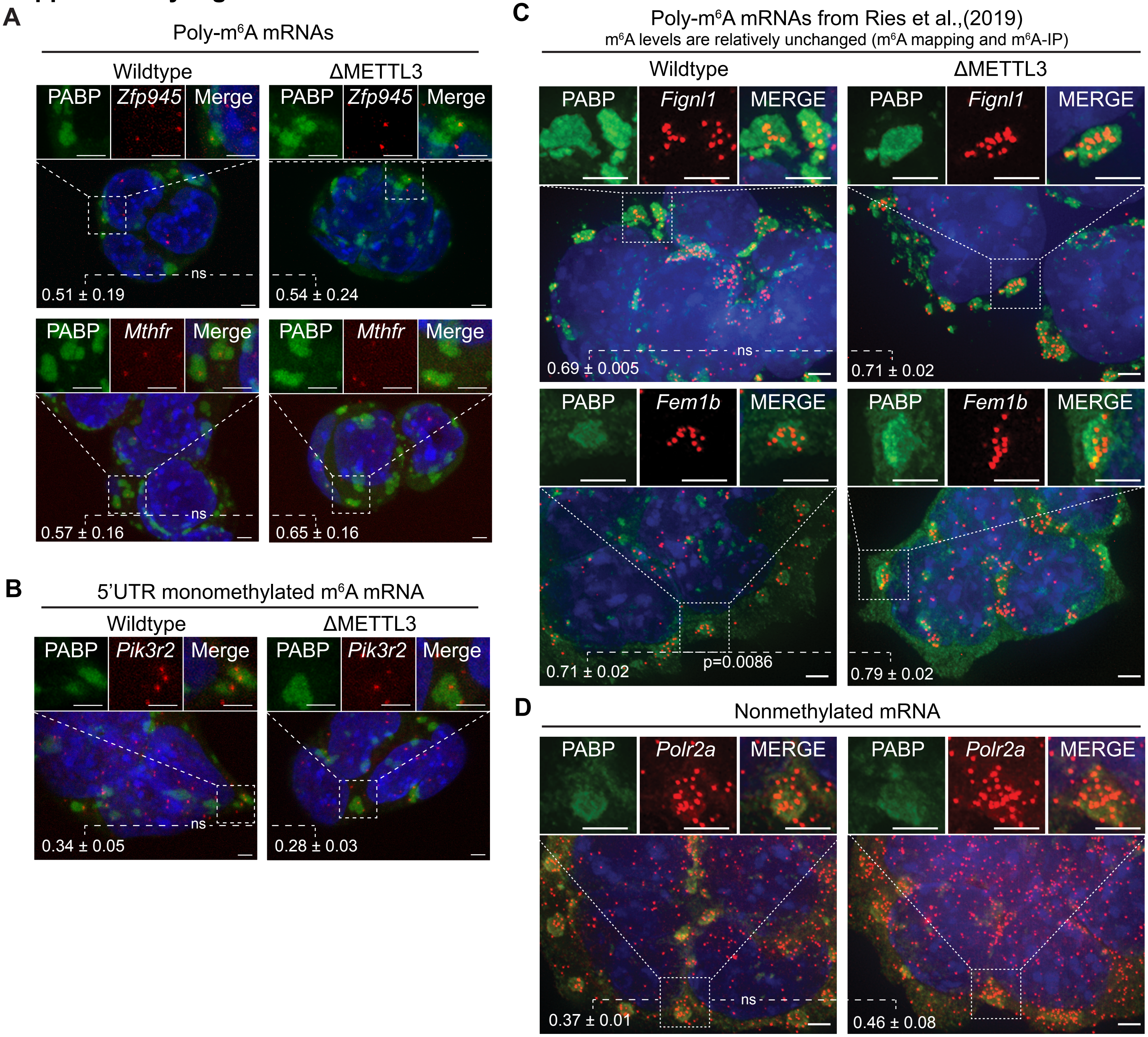

Supplementary Figure 2. There is no difference in the fraction of *Zfp945, Mthfr, Pik3r2, Fignl1 Fem1B,* and *Polr2A* mRNAs in arsenite-induced stress granules between wildtype and Δ*METTL3* mES cells. Representative images of wildtype and ΔMETTL3 mES cells stressed for one hour with arsenite and costained with single-molecule FISH probes against (A) *Zfp945, Mthfr,* (B) *Pike3r2,* (C) *Fignl1, Fem1b,* and (D) *Polr2A* (red) and antibody against PABP protein (green). The nuclei are stained with DAPI (blue). The scale bar is 1 μm for A-B and 3 μm for C-D. Standard deviations are derived from three biological replicates. ns denote not significant respectively (unpaired two-tailed student t-test, $P > 0.05$). Source data are provided for this Figure (see data availability).

Supplementary Figure 3

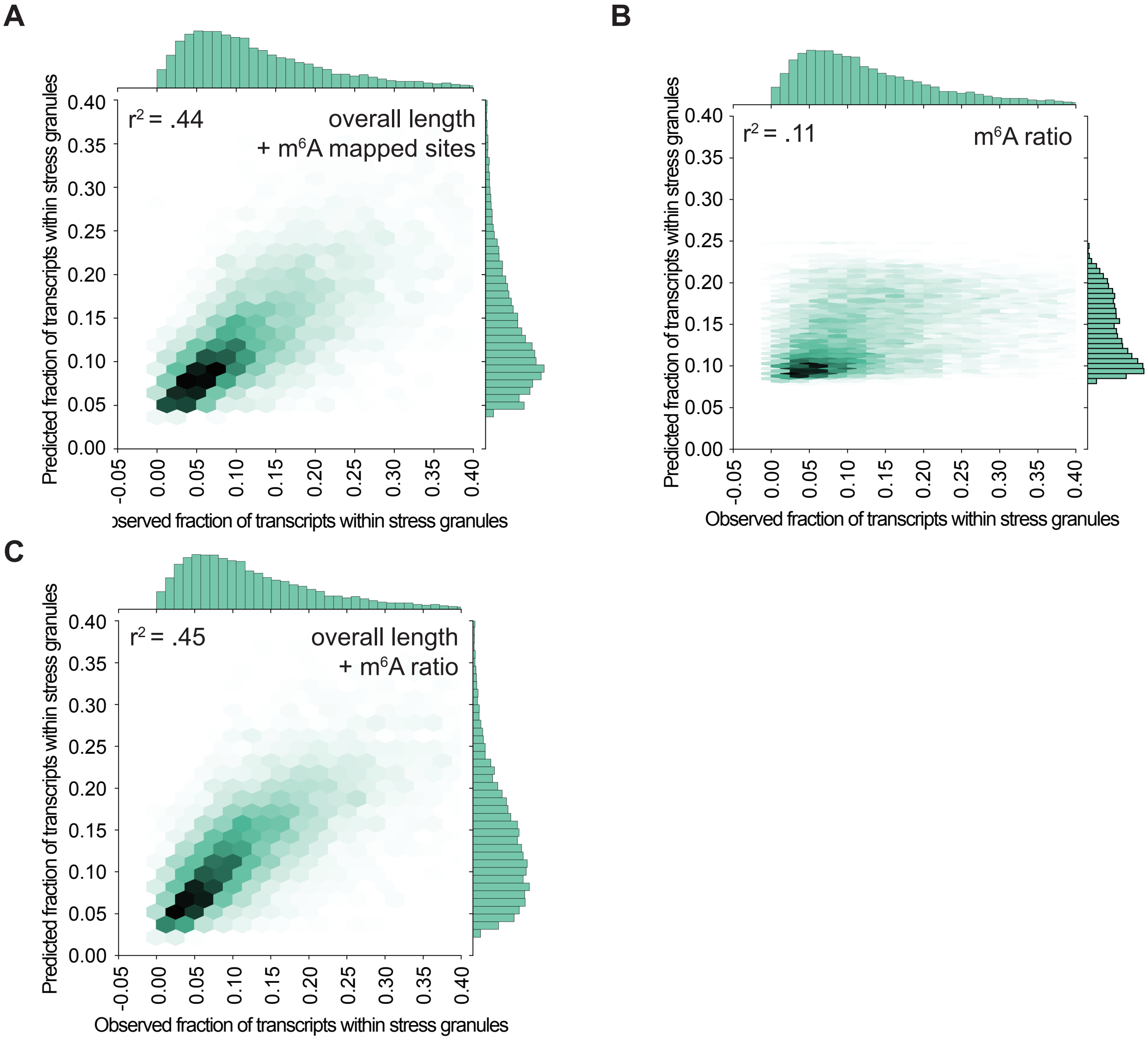

Supplementary Figure 3. Multiple regression analysis between overall transcript length, m^{6}A mapped sites, and m^{6}A ratio on RNA enrichment in stress granules. Scatterplot depicting predicted vs observed fraction of transcripts within stress granules with the following metrics: (A) overall length + m^{6}A mapped sites, (B) m^{6}A ratio, and (C) overall length + m^{6}A ratio. Code data are provided for this Figure (see code availability).
