# Supplementary material for: Limited effects of m6A modification on mRNA partitioning into stress granules
Source: Nat Commun. 2022 Jun 29;13:3735. doi: 10.1038/s41467-022-31358-5 (PMC9243116; doi:10.1038/s41467-022-31358-5)
Supplement: Supplementary file 3 — Description of Additional Supplementary Files [file 41467_2022_31358_MOESM3_ESM.pdf]

Description of Additional Supplementary Files

File Name: Supplementary Movie 1
Description: Z-stack slice movie for wildtype mES cells treated with arsenite and stained for PABP (green) and Aff1 mRNA (red).

File Name: Supplementary Movie 2
Description: Z-stack slice movie for METTL3 KO mES cells treated with arsenite and stained for PABP (green) and Aff1 mRNA (red).

File Name: Supplementary Movie 3
Description: Z-stack slice movie for wildtype mES cells treated with arsenite and stained for PABP (green) and Bahcc1 mRNA (red).

File Name: Supplementary Movie 4
Description: Z-stack slice movie for METTL3 KO mES cells treated with arsenite and stained for PABP (green) and Bahcc1 mRNA (red).

File Name: Supplementary Movie 5
Description: Z-stack slice movie for wildtype mES cells treated with arsenite and stained for PABP (green) and DHX30 mRNA (red).

File Name: Supplementary Movie 6
Description: Z-stack slice movie for METTL3 KO mES cells treated with arsenite and stained for PABP (green) and DHX30 mRNA (red).

File Name: Supplementary Movie 7
Description: Z-stack slice movie for wildtype mES cells treated with arsenite and stained for PABP (green) and Lrp5 mRNA (red).

File Name: Supplementary Movie 8
Description: Z-stack slice movie for METTL3 KO mES cells treated with arsenite and stained for PABP (green) and Lrp5 mRNA (red).

File Name: Supplementary Movie 9
Description: Z-stack slice movie for wildtype mES cells treated with arsenite and stained for PABP (green) and Mtf2 mRNA (red).

File Name: Supplementary Movie 10
Description: Z-stack slice movie for METTL3 KO mES cells treated with arsenite and stained for PABP (green) and Mtf2 mRNA (red).

File Name: Supplementary Movie 11
Description: Z-stack slice movie for wildtype mES cells treated with arsenite and stained for PABP (green) and Mthfr mRNA (red).

File Name: Supplementary Movie 12
Description: Z-stack slice movie for METTL3 KO mES cells treated with arsenite and stained for PABP (green) and Mthfr mRNA (red).

File Name: Supplementary Movie 13
Description: Z-stack slice movie for wildtype mES cells treated with arsenite and stained for PABP (green) and Pik3r2 mRNA (red).

File Name: Supplementary Movie 14
Description: Z-stack slice movie for METTL3 KO mES cells treated with arsenite and stained for PABP (green) and Pik3r2 mRNA (red).

File Name: Supplementary Movie 15
Description: Z-stack slice movie for wildtype mES cells treated with arsenite and stained for PABP (green) and Rictor mRNA (red).

File Name: Supplementary Movie 16
Description: Z-stack slice movie for METTL3 KO mES cells treated with arsenite and stained for PABP (green) and Rictor mRNA (red).

File Name: Supplementary Movie 17
Description: Z-stack slice movie for wildtype mES cells treated with arsenite and stained for PABP (green) and Tnrc6c mRNA (red).

File Name: Supplementary Movie 18
Description: Z-stack slice movie for METTL3 KO mES cells treated with arsenite and stained for PABP (green) and Tnrc6c mRNA (red).

File Name: Supplementary Movie 19
Description: Z-stack slice movie for wildtype mES cells treated with arsenite and stained for PABP (green) and Zeb1 mRNA (red).

File Name: Supplementary Movie 20
Description: Z-stack slice movie for METTL3 KO mES cells treated with arsenite and stained for PABP (green) and Zeb1 mRNA (red).

File Name: Supplementary Movie 21
Description: Z-stack slice movie for wildtype mES cells treated with arsenite and stained for PABP (green) and Zfp945 mRNA (red).

File Name: Supplementary Movie 22
Description: Z-stack slice movie for METTL3 KO mES cells treated with arsenite and stained for PABP (green) and Zfp945 mRNA (red).

File Name: Supplementary Data 1
Description: Data used for analysis for Figures 2 and Supplemental Figure 3

File Name: Supplementary Data 2
Description: Oligos used for single molecule FISH and qRT-PCR primers
