# Supplementary material for: Limited effects of m6A modification on mRNA partitioning into stress granules
Source: Nat Commun. 2022 Jun 29;13:3735. doi: 10.1038/s41467-022-31358-5 (PMC9243116; doi:10.1038/s41467-022-31358-5)
Supplement: Supplementary file 28 — Reporting Summary [file 41467_2022_31358_MOESM28_ESM.pdf]

nature portfolio

Corresponding author(s): Roy Parker

Last updated by author(s): 2022-June-07

Reporting Summary

Nature Portfolio wishes to improve the reproducibility of the work that we publish. This form provides structure for consistency and transparency in reporting. For further information on Nature Portfolio policies, see our Editorial Policies and the Editorial Policy Checklist.

Statistics

For all statistical analyses, confirm that the following items are present in the figure legend, table legend, main text, or Methods section.

n/a	Confirmed	
☐	☒	The exact sample size (*n*) for each experimental group/condition, given as a discrete number and unit of measurement
☐	☒	A statement on whether measurements were taken from distinct samples or whether the same sample was measured repeatedly
☐	☒	The statistical test(s) used AND whether they are one- or two-sided<br>*Only common tests should be described solely by name; describe more complex techniques in the Methods section.*
☒	☐	A description of all covariates tested
☒	☐	A description of any assumptions or corrections, such as tests of normality and adjustment for multiple comparisons
☐	☒	A full description of the statistical parameters including central tendency (e.g. means) or other basic estimates (e.g. regression coefficient) AND variation (e.g. standard deviation) or associated estimates of uncertainty (e.g. confidence intervals)
☐	☒	For null hypothesis testing, the test statistic (e.g. *F*, *t*, *r*) with confidence intervals, effect sizes, degrees of freedom and *P* value noted<br>*Give P values as exact values whenever suitable.*
☒	☐	For Bayesian analysis, information on the choice of priors and Markov chain Monte Carlo settings
☒	☐	For hierarchical and complex designs, identification of the appropriate level for tests and full reporting of outcomes
☐	☒	Estimates of effect sizes (e.g. Cohen's *d*, Pearson's *r*), indicating how they were calculated

Our web collection on statistics for biologists contains articles on many of the points above.

Software and code

Policy information about availability of computer code

Data collection	softWoRx (6.5.2), NIS-Elements AR (5.30.05)
Data analysis	Fiji ImageJ (1.51s)<br>Imaris (9.7.0)<br>Sci-kit learn version 1.1.1

For manuscripts utilizing custom algorithms or software that are central to the research but not yet described in published literature, software must be made available to editors and reviewers. We strongly encourage code deposition in a community repository (e.g. GitHub). See the Nature Portfolio guidelines for submitting code & software for further information.

Data

Policy information about availability of data

All manuscripts must include a data availability statement. This statement should provide the following information, where applicable:

- Accession codes, unique identifiers, or web links for publicly available datasets
- A description of any restrictions on data availability
- For clinical datasets or third party data, please ensure that the statement adheres to our policy

Source data are provided with this paper. Quantitative source data for Figures 1, 2A, 2E, 2F, 3B, 3D, Supplementary Figures 1A-D, and Supplementary Figure 2A-D in

this study are provided as an excel Source Data file. Quantitative source data for Figures 2B-D and Supplementary Figure 3 are deposited at https://github.com/tmatheny/m6a/. Raw imaging files that were included in the figures are deposited at Figshare.com https://figshare.com/articles/dataset/Raw_images_for_Figure_1_Figure_3_and_Supplementary_Figure_2_zip/19780576/. Not all raw imaging files were included due to number and sizes of images. These raw imaging files can be shared upon request.

Human research participants

Policy information about studies involving human research participants and Sex and Gender in Research.

Reporting on sex and gender	*Use the terms sex (biological attribute) and gender (shaped by social and cultural circumstances) carefully in order to avoid confusing both terms. Indicate if findings apply to only one sex or gender; describe whether sex and gender were considered in study design whether sex and/or gender was determined based on self-reporting or assigned and methods used. Provide in the source data disaggregated sex and gender data where this information has been collected, and consent has been obtained for sharing of individual-level data; provide overall numbers in this Reporting Summary. Please state if this information has not been collected. Report sex- and gender-based analyses where performed, justify reasons for lack of sex- and gender-based analysis.*
Population characteristics	*Describe the covariate-relevant population characteristics of the human research participants (e.g. age, genotypic information, past and current diagnosis and treatment categories). If you filled out the behavioural & social sciences study design questions and have nothing to add here, write "See above."*
Recruitment	*Describe how participants were recruited. Outline any potential self-selection bias or other biases that may be present and how these are likely to impact results.*
Ethics oversight	*Identify the organization(s) that approved the study protocol.*

Note that full information on the approval of the study protocol must also be provided in the manuscript.

Field-specific reporting

Please select the one below that is the best fit for your research. If you are not sure, read the appropriate sections before making your selection.

☒ Life sciences ☐ Behavioural & social sciences ☐ Ecological, evolutionary & environmental sciences

For a reference copy of the document with all sections, see nature.com/documents/nr-reporting-summary-flat.pdf

Life sciences study design

All studies must disclose on these points even when the disclosure is negative.

Sample size	Supplemental Figure 1ai) n = 2 standard curve<br>Supplemental Figure 1aii) n = 2 from two biology replicates (samples)<br>Supplemental Figure 1b) Analysis from m6A mapping data collected by Batista et al., (2014) Cell Stem Cell<br>Supplemental Figure 1c) qRT-PCR ratios are derived from two biological replicates of m6A-IP of WT and METTL3 KO cells<br>Supplemental Figure 1d) qRT-PCR values are derived from two biological replicates of WT and METTL3 KO cells<br>Supplemental Figure 2A-D) Three biological replicates<br>Figure 1) Three biological replicates<br>Figure 2) Data was reanalyzed from preexisting datasets collected by Khong et al., (2017) Mol Cell, Xiang et al., (2017) Nature, and Molinie et al., (2016). Transcript lengths were obtained from pybiomart.<br>Figure 3b) n = 45, 29, 25, and 13 cells for λN, G3BP1-λN, YTHDF1-λN, and YTHDF2-λN respectively.<br>Figure 3bii) n = 21,33,37, and 25 cells for λN, G3BP1-λN, YTHDF1-λN, and YTHDF2-λN respectively.<br>Figure 3c) Representative images shown from analysis in Figure 3d.<br>figure 3di) n = 45, 29, 25, and 13 cells for λN, G3BP1-λN, YTHDF1-λN, and YTHDF2-λN respectively.<br>Figure 3dii) n = 21,33,37, and 25 cells for λN, G3BP1-λN, YTHDF1-λN, and YTHDF2-λN respectively.
Data exclusions	In figure 3, we excluded cells that are expressing two little or too much λN, G3BP1-λN, YTHDF1-λN, and YTHDF2-λN. If it was expressed too little, it's unclear if sufficient proteins will be tethered to the reporter. If it was expressed too much, we discovered that it can form spontaneous stress granules in the absense of stress. We selected a specific window of total fluoresence. This was noted in materials and methods. Our criteria is 50,000 < Cell Total Cell Fluorescence < 2,000,000 (determined by ImageJ)
Replication	Reproducibility was confirmed with at least 3 independent experimental replicates for each assay except Supplemental Figures 1. The mettl3 knockout mES cell line was previously confirmed to be m6A depleted in Batista et al., (2015) Cell Stem Cell. These experiments were to double check that it the cells that we received from Dr. Batista are indeed m6A depleted.
Randomization	This is not relevant to our study because we used cell culture systems which are largely homoegenous.
Blinding	To count fish spots inside and outside stress granules in a unbiased manner, we blinded the analysis by altering the file names to a numeric number (Supplemental Figure 2C,D and 3). Figure 1 and Supplemental Figure 2A,B are not applicable since we used the same code from

imaging software to analyze the data consistently. Blinding is also not relevant in all other experiments (qRT-PCR etc) because the approaches /analysis used are applied equally to all the samples that were directly compared.

Reporting for specific materials, systems and methods

We require information from authors about some types of materials, experimental systems and methods used in many studies. Here, indicate whether each material, system or method listed is relevant to your study. If you are not sure if a list item applies to your research, read the appropriate section before selecting a response.

Materials & experimental systems

n/a	Involved in the study
☐	☒ Antibodies
☐	☒ Eukaryotic cell lines
☒	☐ Palaeontology and archaeology
☒	☐ Animals and other organisms
☒	☐ Clinical data
☒	☐ Dual use research of concern

Methods

n/a	Involved in the study
☒	☐ ChIP-seq
☒	☐ Flow cytometry
☒	☐ MRI-based neuroimaging

Antibodies

Antibodies used

mouse α-G3BP primary antibody (ab56574; Abcam), Rabbit α-PABP primary antibody (ab21060; Abcam), goat α-mouse FITC-conjugated secondary antibody (ab6785; Abcam), donkey anti-rabbit Alexa-fluor 555 antibody (ab150062, Abcam), and rabbit m6A antibody (202 003, Synaptic Systems)

Validation

Manufacturer's website ab56574: https://www.abcam.com/g3bp-antibody-ab56574.html
Manufacturer's website: ab21060: https://www.abcam.com/pabp-antibody-ab21060.html
Manufacturer's website: ab6785: https://www.abcam.com/goat-mouse-igg-hl-fitc-ab6785.html
Manufacturer's website: ab150062: https://www.abcam.com/donkey-rabbit-igg-hl-alexa-fluor-555-preadsorbed-ab150062.html
Manufacturer's website: 202 003: https://sysy.com/product/202003

Eukaryotic cell lines

Policy information about cell lines and Sex and Gender in Research

Cell line source(s)

U-2 OS cells are from Dr. Nancy Kedersha and Dr. Paul Anderson (Brigham and Women's Hospital). Wildtype and METTL3 KO mES cells are from Dr. Pedro Batista (NIH). HEK293T cells are from Christopher Sullivan (UT Austin)

Authentication

None of the cell lines were authenticated

Mycoplasma contamination

Cell lines were not tested for mycoplasm contamination

Commonly misidentified lines
(See ICLAC register)

No commonly misidentified lines were used.
